# Inter-Relationship between Testicular Dysgenesis and Leydig Cell Function in the Masculinization Programming Window in the Rat

**DOI:** 10.1371/journal.pone.0030111

**Published:** 2012-01-11

**Authors:** Sander van den Driesche, Petros Kolovos, Sophie Platts, Amanda J. Drake, Richard M. Sharpe

**Affiliations:** 1 MRC Centre for Reproductive Health, The University of Edinburgh, Edinburgh, United Kingdom; 2 Endocrinology Unit, University/BHF Centre for Cardiovascular Science, The University of Edinburgh, Edinburgh, United Kingdom; Clermont Université, France

## Abstract

The testicular dysgenesis syndrome (TDS) hypothesis proposes that maldevelopment of the testis, irrespective of cause, leads to malfunction of the somatic (Leydig, Sertoli) cells and consequent downstream TDS disorders. Studies in rats exposed *in utero* to di(*n*-butyl) phthalate (DBP) have strongly supported the TDS concept, but so far no direct evidence has been produced that links dysgenesis *per se* to somatic cell dysfunction, in particular to androgen production/action during the ‘masculinization programming window’ (MPW; e15.5–e18.5). Normal reproductive tract development and anogenital distance (AGD) are programmed within the MPW, and TDS disorders arise because of deficiencies in this programming. However, DBP-induced focal testicular dysgenesis (Leydig cell aggregation, ectopic Sertoli cells, malformed seminiferous cords) is not evident until after the MPW. Therefore, we used AGD as a read-out of androgen exposure in the MPW, and investigated if this measure was related to objectively quantified dysgenesis (Leydig cell aggregation) at e21.5 in male fetuses exposed to vehicle, DBP (500 or 750 mg/kg/day) or the synthetic glucocorticoid dexamethasone (Dex; alone or plus DBP-500) from e15.5–e18.5 (MPW), e13.5–e20.5 or e19.5–e20.5 (late window). Dysgenesis was found only in animals exposed to DBP during the MPW, and was negatively correlated (R^2^ = −0.5) with AGD at e21.5 and at postnatal day 8, irrespective of treatment period. Dysgenesis was also negatively correlated (R^2^ = –0.5) with intratesticular testosterone (ITT) at e21.5, but only when treatments in short windows (MPW, late window) were excluded; the same was true for correlation between AGD and ITT. We conclude that AGD, reflecting Leydig cell function solely within the MPW, is strongly related to focal dysgenesis. Our results point to this occurring because of a common early mechanism, targeted by DBP that determines both dysgenesis and early (during the MPW) fetal Leydig cell dysfunction. The findings provide strong validation of the TDS hypothesis.

## Introduction

Cryptorchidism, hypospadias, low sperm count and testicular germ cell cancer are disorders of male reproductive health that have a high or increasing incidence in the Western world [1], [2]. These disorders have been hypothesized to comprise a testicular dysgenesis syndrome (TDS) with a common fetal origin [3], [4]. This hypothesis proposes that maldevelopment of the testis, which could have numerous primary causes, leads secondarily to malfunction of the Leydig and/or Sertoli cells and consequent downstream TDS disorders [1], [3], [4]. There has been considerable interest in identifying the mechanistic origins of TDS disorders and the events that lead to their development. As these fetal events are impossible to study in humans, animal models have been developed, such as *in utero* exposure of pregnant rats to di(*n*-butyl) phthalate (DBP), to try and investigate the mechanisms that underlie TDS disorders [5], [6], [7]. These studies have produced strong supporting evidence for the relationship between somatic cell dysfunction and TDS disorders [8], [9], [10], [11].

**Figure 1:**
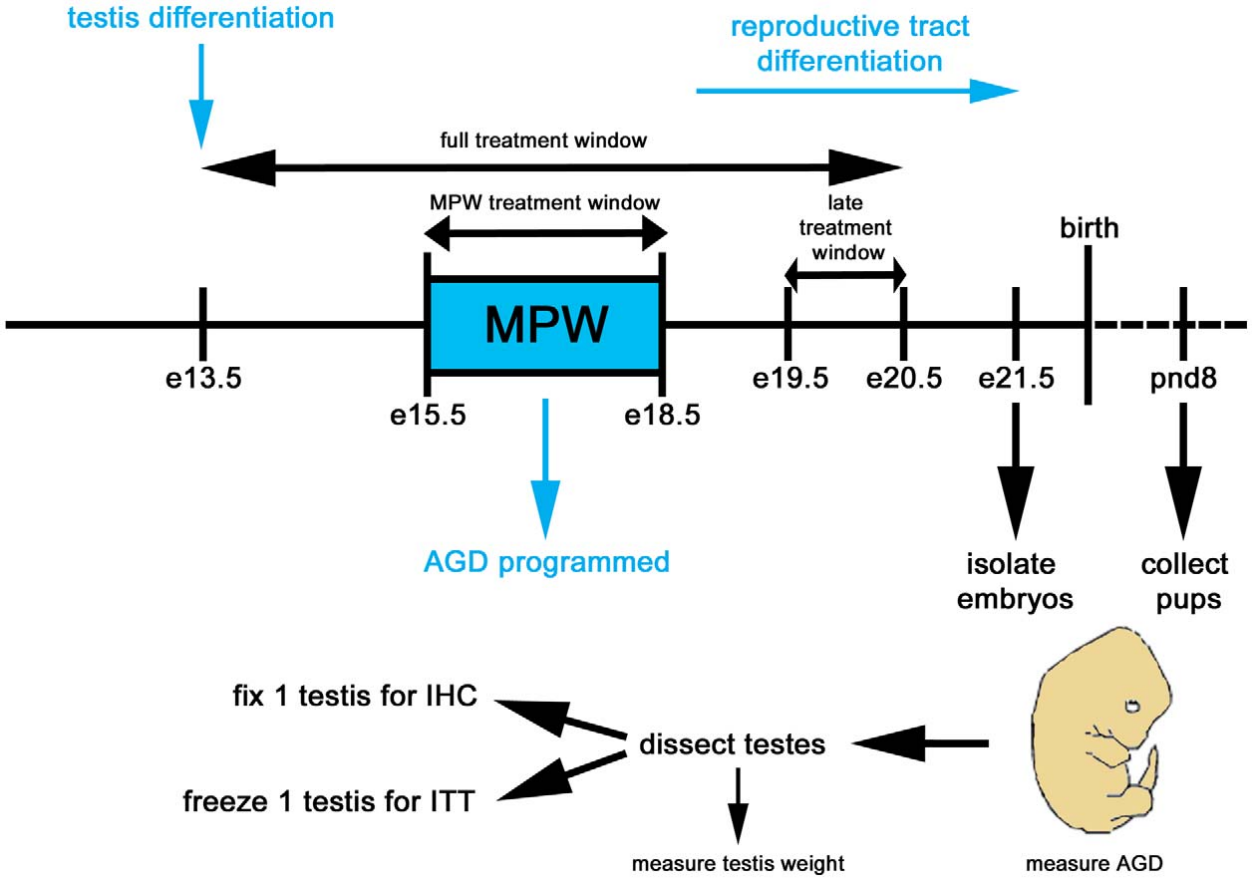
Schematic representation of the various treatment windows and experimental design. Also indicated in blue are testis differentiation in the rat (at ∼embryonic day (e)13.5), reproductive tract differentiation (from ∼e18.5 onwards) and the masculinization programming window (MPW, e15.5–e18.5), during which anogenital distance (AGD) is programmed. Three treatment windows were used in this study, namely ‘full treatment window’ (e13.5–e20.5), ‘MPW treatment window’ (e15.5–e18.5) and ‘late treatment window’ (e19.5–e20.5). At e21.5 embryos were isolated. For each embryo, AGD was measured, testes were dissected and weighed, before 1 testis was fixed for subsequent immunohistochemistry (IHC) and 1 testis was frozen on dry ice for subsequent intratesticular testosterone (ITT) measurement. In a separate experiment, pregnant rats were treated from e13.5–e21.5 and pups were collected on postnatal day (pnd) 8 and the same procedures as for the fetal samples were undertaken.

An important finding in relation to TDS disorders is that androgen action during a ‘masculinization programming window’ (MPW; e15.5–e18.5 in rats) is essential for setting up normal reproductive tract development and masculinization of anogenital distance (AGD; Fig. 1) [12], [13], [14], [15]. Insufficient androgen exposure during the MPW, for example as the result of exposure to certain endocrine disrupting compounds [10], leads to smaller adult reproductive organ size (testes, prostate, seminal vesicles, penis) and increased risk of reproductive disorders (cryptorchidism, hypospadias), as well as a reduced AGD [14], [15]. AGD is sexually dimorphic in rodents [16], [17] and humans [18], [19], [20], and toxicologists have long used AGD as an index of overall fetal androgen exposure [17], [21]. In humans the MPW is postulated to occur within the period ∼8–14 weeks' gestation [15]. As in the rat, shorter AGD is associated with occurrence of hypospadias, cryptorchidism and shorter penis length at birth [22], [23] and with low sperm counts and infertility in adulthood in humans [24], [25], suggesting that AGD could also be used as a non-invasive ‘read-out’ of *in utero* fetal androgen action/exposure (during the MPW) in newborn boys.

*In utero* exposure of rats to DBP induces testicular changes remarkably similar to TDS in humans, including the induction of focal areas of dysgenesis in otherwise normal testes [5], [26], [27], [28], [29]. Therefore, this model can potentially be used to dissect the mechanisms that underlie dysgenesis and, importantly, the inter-relationship of dysgenesis to somatic cell function, notably that of the fetal Leydig cells. The latter is especially important because we have shown there is a temporal ‘mis-match’ between DBP-induced inhibition of Leydig cell steroidogenesis in the MPW, which underlies TDS disorders [14], [15] and the occurrence of focal testicular dysgenesis (malformed seminiferous cords, Leydig cell aggregation, intratubular Leydig cells), which is not evident until after the MPW [8], [9]. One hallmark of DBP-induced dysgenesis is the abnormal formation of large Leydig cell aggregates in central regions of the fetal rat testis, which can be objectively quantified [8]. In the present studies we used this hallmark, in combination with DBP exposure in various fetal time windows, to determine the inter-relationships in late gestation (e21.5) between dysgenesis and steroidogenic function, earlier in the MPW (as indicated by AGD measurement), and currently by measurement of intratesticular testosterone at e21.5. We also included a treatment, dexamethasone (Dex), that modestly reduces Leydig cell steroidogenic function and AGD, but does not cause detectable dysgenesis when administered on its own but exacerbates the endocrine effects of DBP [30]. Our results suggest that impaired Leydig cell function in the MPW and the degree of subsequent dysgenesis are inter-related, supporting the idea that both features have a common origin, in keeping with the TDS hypothesis.

## Results

### DBP-induction of focal testicular dysgenesis (fetal Leydig cell aggregation)

**Figure 2:**
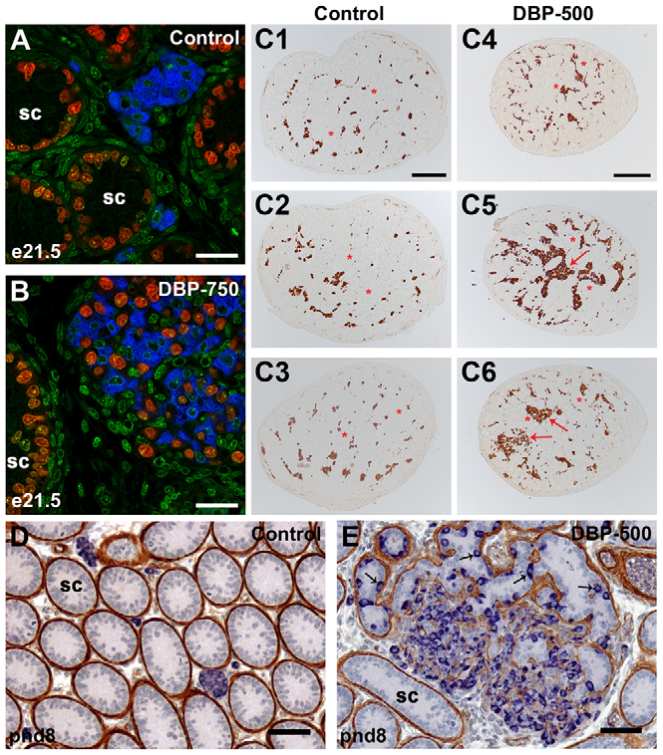
Immunohistological analysis of focal dysgenetic areas in rat testes exposed to vehicle (control) or dibutyl phthalate (DBP). (A–B) Double immunofluorescence for 3β-HSD (blue) and Sox-9 (red) on e21.5 testis sections from (A) vehicle (control) and (B) DBP-exposed (750 mg/kg/) animals, illustrating focal dysgenesis in which Leydig cell aggregates contain ectopically localized Sertoli cells. Green depicts DAPI nuclear counterstain. SC = seminiferous cords. Scale bar = 20 µm. (C1–C6) Example of sections stained for 3β-HSD (brown) used for Leydig cell aggregate analysis (see Figure 3). Arrows indicate large Leydig cell aggregates, asterisks indicate seminiferous cords. Scale bar = 200 µm. (D–E) Double immunohistochemistry for 3β-HSD (blue) and SMA (brown) on postnatal day (pnd) 8 testis sections from (D) vehicle (control) and (E) DBP-exposed (500 mg/kg/) animals, illustrating focal dysgenesis after DBP-exposure, with large Leydig cell aggregates and malformed seminiferous cords and intratubular Leydig cells (arrows). Scale bar = 50 µm.

**Figure 3:**
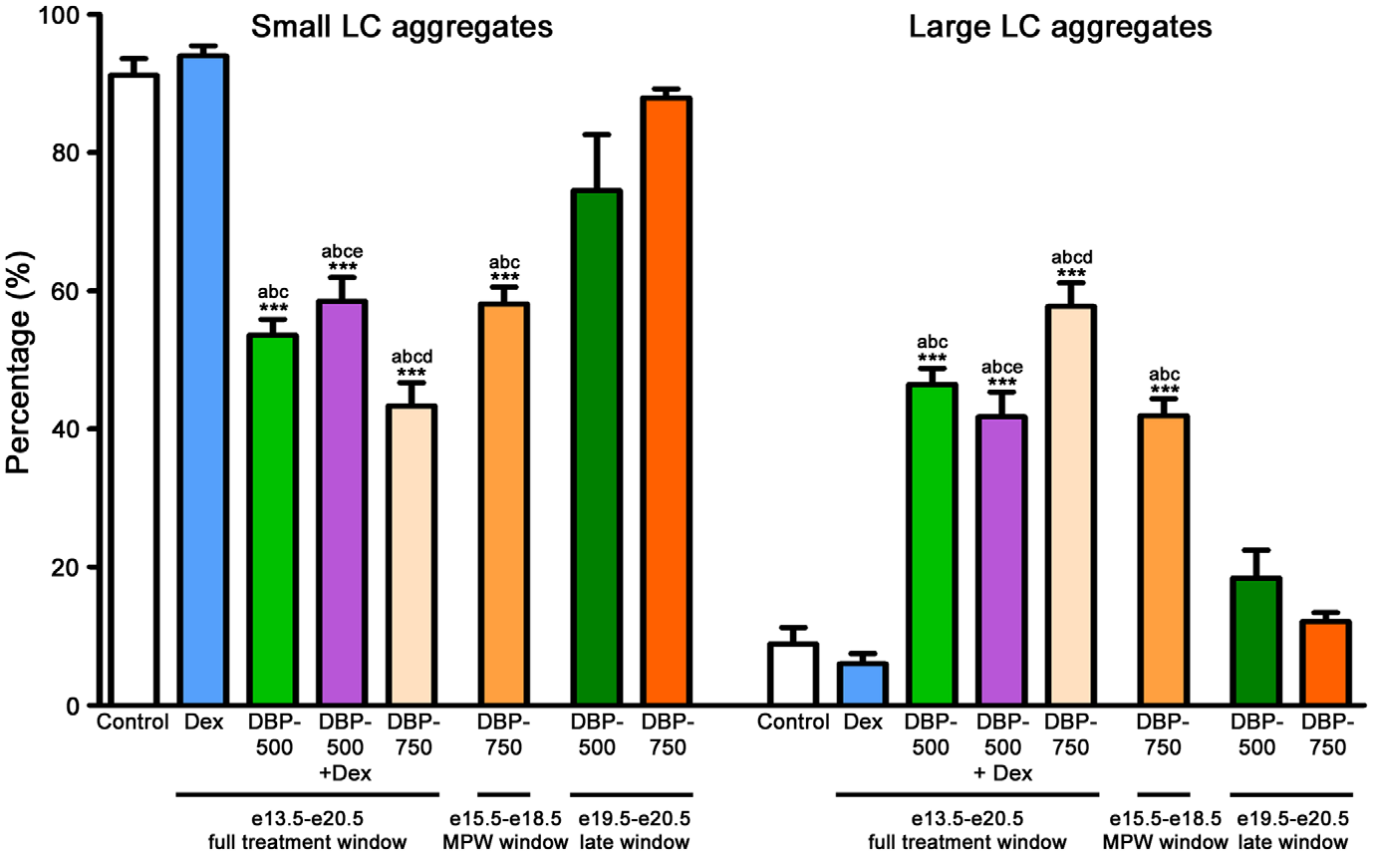
Contribution of small and large Leydig cell aggregates to the total Leydig cell aggregate area per testis in e21.5 rat testes after *in utero* exposure to vehicle (control) or dibutyl phthalate (DBP-500 or 750 mg/kg), dexamethasone (Dex 100 µg/kg) or DBP-500+Dex from e13.5–e20.5 (full treatment window), e15.5–e18.5 (MPW window) or e19.5–e20.5 (late window). Values are Means ± SEM for 8–15 animals from 3–5 litters per treatment group. ***p<0.001, in comparison with controls; ^a^p<0.001 in comparison with Dex group (except p<0.05 when Dex is compared with DBP-500 late window treatment); ^b^p<0.05 in comparison with DBP-500 late window group; ^c^p<0.001 in comparison with DBP-750 late window group; ^d^p<0.01 in comparison with DBP-750 MPW window group; ^e^p<0.05 in comparison with DBP-750 full treatment window group.

Exposure of animals to a high dose of DBP (750 mg/kg/day) from e13.5–e20.5 induced focal dysgenetic areas at e21.5 in which abnormal Leydig cell aggregates were intermingled with ectopic Sertoli cells (ie outside of seminiferous cords) (Fig. 2A, B). By postnatal day 8, mis-shapen seminiferous cords form within these areas and intratubular Leydig cells are found (Fig. 2E), neither of which are found in controls (Fig. 2D). Leydig cell aggregation at e21.5 was therefore analyzed as a measure of focal testicular dysgenesis, using previously established methods [8]. Three sections per e21.5 testis were immunostained with 3β-HSD before being analyzed using stereology (Fig. 2C1–C6). The measured area of each Leydig cell aggregate was then expressed as a percentage of the total Leydig cell area in that section as a means of quantifying the degree of aggregation (Leydig cell number per testis is unchanged; [8]). A significantly higher percentage of Leydig cells were present in large aggregates in animals exposed to 500 mg/kg DBP (DBP-500), 750 mg/kg DBP (DBP-750), DBP-500+Dex during the full treatment window (e13.5–e20.5) or during the MPW (e15.5–e18.5), when compared with vehicle-exposed controls (Fig. 3). No significant effect on Leydig cell aggregation was observed when animals were exposed to Dex on its own or to DBP-500 or DBP-750 when these were administered during the late treatment window (e19.5–e20.5; ie after the MPW) (Fig. 3). We also analyzed the data using litter means and similar results were found (Fig. S1).

### Effects of the different treatments and treatment windows on AGD and intratesticular testosterone at e21.5

**Figure 4:**
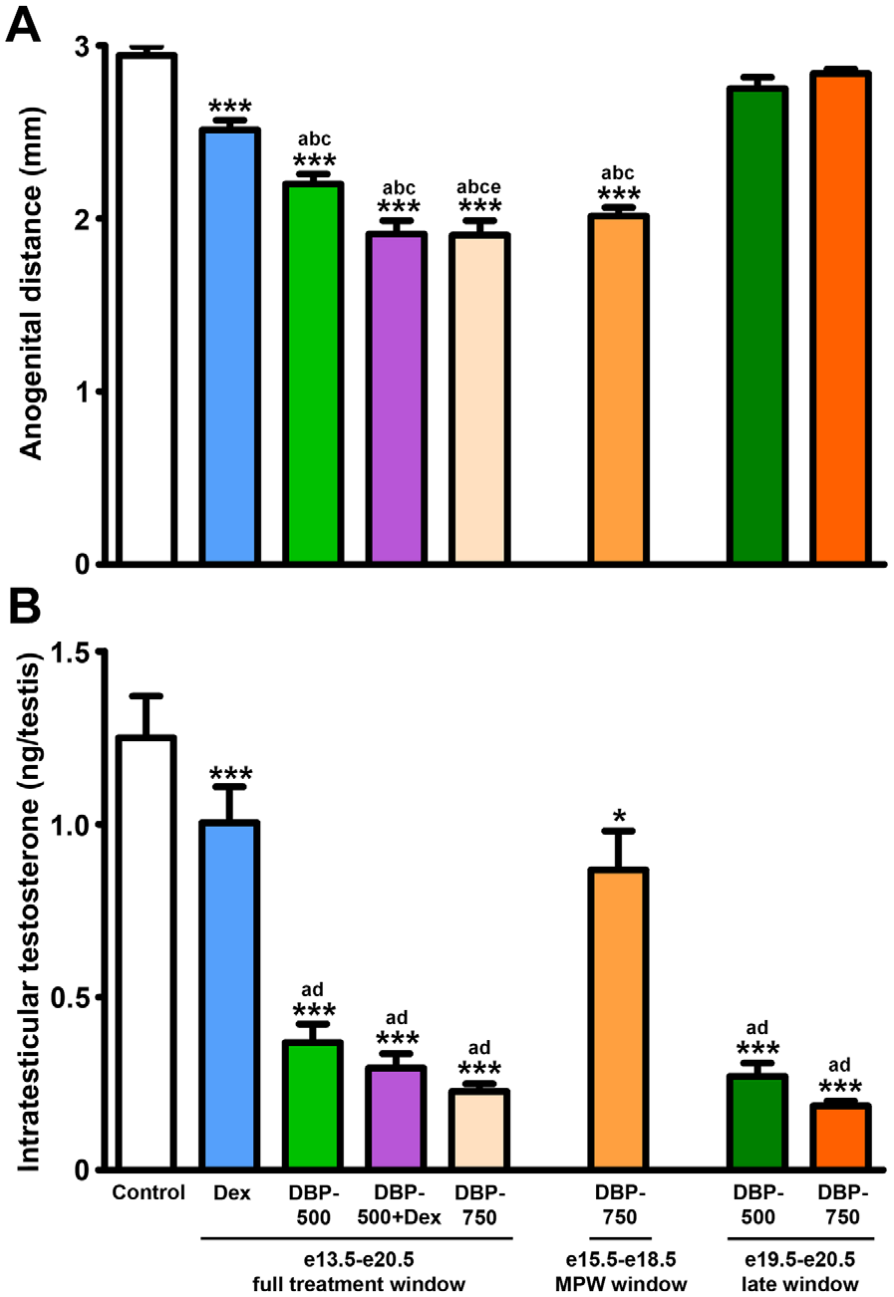
Anogenital distance (AGD) and intratesticular testosterone (ITT) in rats at e21.5 after *in utero* exposure to vehicle (control), dibutyl phthalate (DBP-500 or 750 mg/kg), dexamethasone (Dex 100 µg/kg) or DBP-500+Dex from e13.5–e20.5 (full treatment window), e15.5–e18.5 (MPW window) or e19.5–e20.5 (late window). Only treatments which included the masculinization programming window (MPW) resulted in a significant reduction in AGD in animals (A), whereas ITT was maximally reduced when treatment included the late (e19.5–e20.5) window (B). Values are Means ± SEM for 18–39 animals from 3–7 litters per group. ***p<0.001, in comparison with controls; ^a^p<0.001 in comparison with Dex group; ^b^p<0.001 in comparison with DBP-500 late window group; ^c^p<0.001 in comparison with DBP-750 late window group; ^d^p<0.01 in comparison with DBP-750 MPW window group; ^e^p<0.05 in comparison with DBP-500 full treatment window group.

Fetal exposure to Dex, DBP-500, DBP-750 or DBP-500+Dex from e13.5–e20.5 all significantly reduced AGD at e21.5 (Fig. 4A), indicative of reduced androgen production/exposure during the MPW (e15.5–e18.5). Exposure to DBP-750 just during the MPW also significantly reduced AGD at e21.5, whereas exposure to DBP-500 or DBP-750 after the MPW (from e19.5–e20.5 = late treatment window) did not alter AGD at e21.5 when compared with vehicle-exposed controls (Fig. 4A), confirming that AGD is programmed only by androgen action during the MPW [15], [31]. In contrast, intratesticular testosterone (ITT) at e21.5 was reduced equally when animals were exposed to DBP-500 or DBP-750 from e13.5–e20.5 or during the late treatment window (e19.5–e20.5) (Fig. 4B). When animals were exposed to DBP-750 only during the MPW, only a modest reduction in ITT was still evident at e21.5 (Fig. 4B), confirming that DBP-induced reduction of ITT largely recovers once treatment ceases (i.e. at e18.5) [31]. When data was analyzed using litter means similar results were found, except for effects of exposure to Dex on AGD and ITT (Fig. S2).

### Relationship between dysgenesis (Leydig cell aggregation) and AGD and intratesticular testosterone (ITT) at e21.5

**Figure 5:**
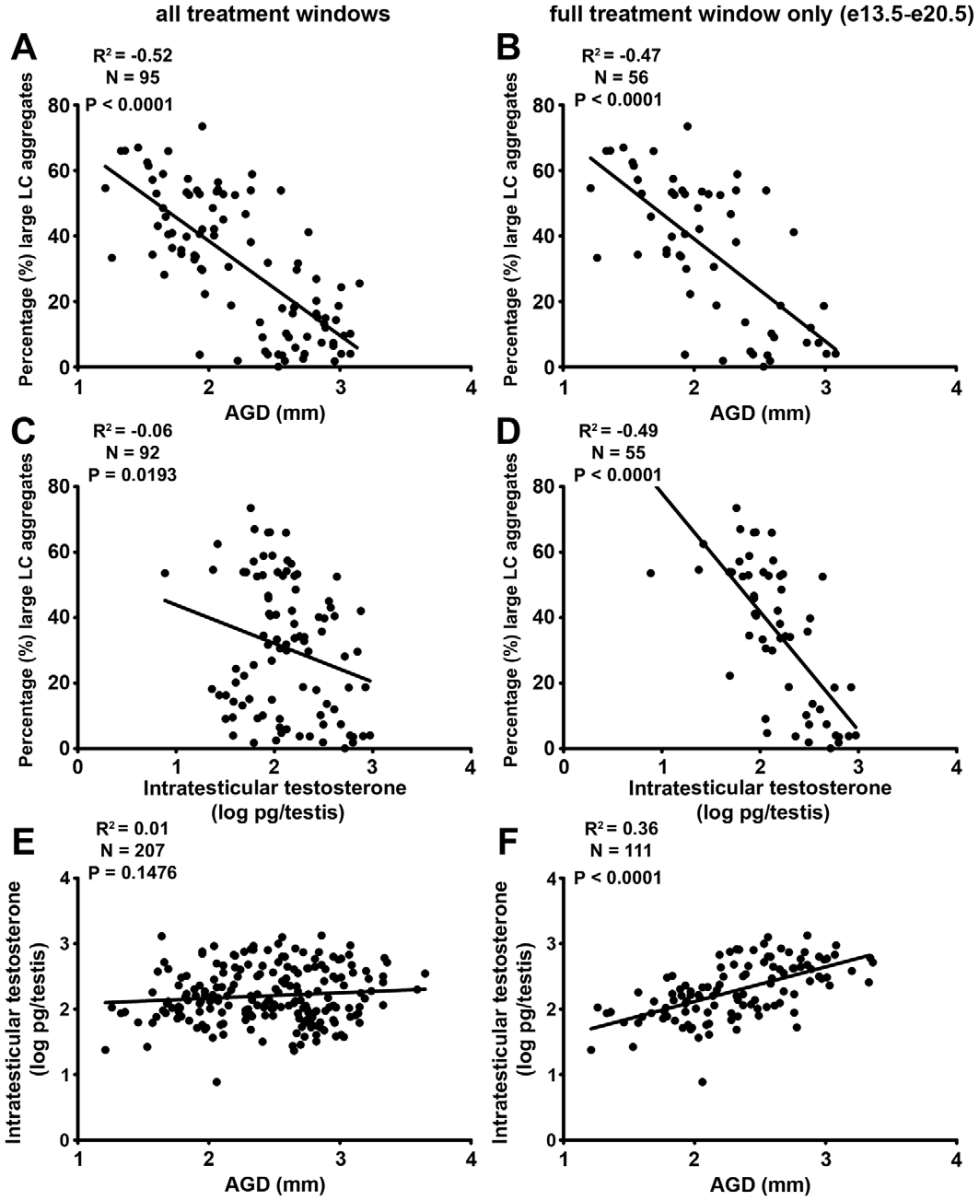
Relationship between Leydig cell (LC) aggregation ( = focal dysgenesis) and anogenital distance (AGD) (A, B) or intratesticular testosterone (ITT) at e21.5 (C, D) or between AGD and ITT at e21.5 (E, F) in animals exposed *in utero* to vehicle (control), dibutyl phthalate (DBP-500 or 750 mg/kg), dexamethasone (Dex 100 µg/kg) or DBP-500+Dex during all treatment windows (A, C, E), or during the full treatment window (e13.5–e20.5) only (B, D, F). Dysgenesis is negatively correlated with AGD irrespective of treatment period (A, B) whereas all other correlations were affected by the treatment period (full details in text).

To establish if Leydig cell aggregation (reflecting focal testicular dysgenesis later in gestation) was correlated with AGD (reflecting androgen production/action during the MPW), these measures were compared for all animals from all treatment groups or after excluding animals that were only exposed during the MPW or during the late time window. Both analyses showed that Leydig cell aggregation was strongly negatively correlated with AGD at e21.5 (Fig. 5A, B; R^2^ = −0.5, P<0.0001). A similar analysis undertaken between Leydig cell aggregation and ITT at e21.5 still showed a significant negative relationship, but this was far stronger (R^2^ = −0.5, P<0.0001; Fig. 5D) for animals exposed to DBP ± Dex throughout the period e13.5–e20.5, than when this analysis also included animals exposed to DBP only in the MPW or in the late window (R^2^ = −0.06, P = 0.02; Fig. 5C). Consistent with these results, analysis of AGD versus ITT at e21.5 revealed no significant association (P>0.1) when data for all treatment groups were included (Fig. 5E) but a significant positive correlation (R^2^ = 0.36, P<0.0001) when animals that were only exposed during the MPW or during the late time window were excluded (Fig. 4F). However, even in the latter instance, the slope of the regression line was shallow when compared with that relating Leydig cell aggregation to AGD (Fig. 5A, B, D). Analyzing the data using litter means showed the same results, with even stronger correlations (Fig. S3).

### Fetal Leydig cell aggregation at postnatal day 8 and its correlation with AGD

**Figure 6:**
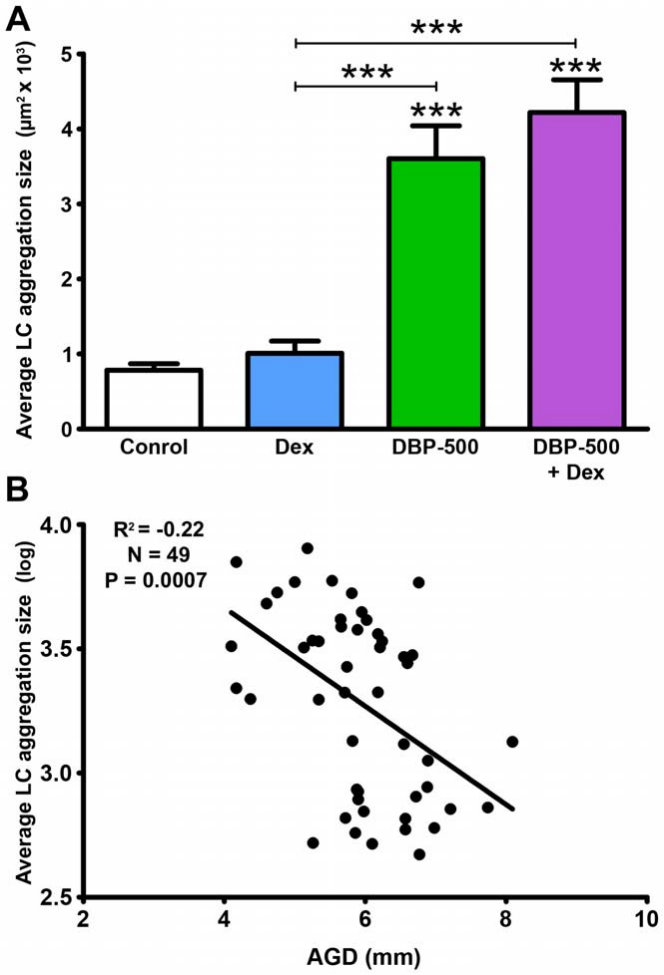
Relationship between Leydig cell (LC) aggregation ( = focal dysgenesis) and anogenital distance (AGD) at postnatal day (pnd) 8 after *in utero* exposure to vehicle (control), dibutyl phthalate (DBP-500 mg/kg), dexamethasone (Dex 100 µg/kg) or DBP-500+Dex from e13.5–e21.5. (A) Average size of the 3 largest Leydig cell aggregates is shown as Means ± SEM for 8–17 animals from 3–6 litters. ***p<0.001, in comparison with controls; other comparisons are indicated by capped lines. (B) Correlation between dysgenesis and anogenital distance (AGD) in pnd8 animals.

For some treatment groups (Dex, DBP-500 or DBP-500+Dex) involving exposure of animals from e13.5–e21.5, dams were allowed to give birth and the pups were then culled at postnatal day 8 (pnd8) to investigate whether the treatment effects on Leydig cell aggregation and AGD evident at e21.5, and their correlation, persisted after birth and cessation of treatment. Pnd8 is about the latest time point at which fetal Leydig cells can still be easily discerned in the rat testis, but their widespread distribution and small numbers meant that a different method had to be used for measuring their aggregation, as explained in Materials & Methods. At pnd8, pups exposed to DBP ± Dex, but not to Dex alone, still exhibited marked evidence of Leydig cell aggregation (Fig. 6A), and this was still negatively correlated with AGD (Fig. 6B) as found before birth (Fig. 5).

## Discussion

The testicular dysgenesis syndrome (TDS) hypothesis proposes that maldevelopment of the testis, which could have numerous primary causes, leads to malfunction of the Leydig and/or Sertoli cells and consequent downstream TDS disorders [4]. Previous studies have demonstrated the importance of a ‘masculinization programming window’ (MPW; e15.5–e18.5) in rats, during which sufficient androgen action is essential for laying the foundations of correct development of the male reproductive tract [10], [15]. Experimental studies in animals have shown that when androgen production or action is manipulated during the MPW in rats, a higher incidence of TDS-like disorders is observed in male offspring [10], [15], [30], [32]. Moreover, DBP exposure results in the occurrence in some animals of focal areas of dysgenesis in the testis, although these are not evident until late in gestation (after the MPW) or after birth (malformed seminiferous cords, intratubular Leydig cells) [33]. Therefore, the connection between dysgenesis and somatic (Leydig) cell dysfunction in the MPW is unclear. The present studies demonstrate this connection by showing that occurrence of dysgenesis in individual animals is inversely related to AGD, which provides a measure of androgen exposure in the MPW, and thus of somatic (Leydig) cell function in this critical period.

In order to analyze focal dysgenesis, we quantified the level of Leydig cell aggregation by following the same procedures as used previously in our laboratory [8]. Because this method uses three sections at approximately 25, 50, and 75% intervals through the serially sectioned testis, it provides an objective quantification of the level of Leydig cell aggregation at the whole testis level. We validated this method to demonstrate a decrease in small Leydig cell aggregates and an increase in large Leydig cell aggregates after *in utero* exposure to DBP, as previously described [8]. Using this method we then demonstrated that large Leydig cell aggregates were found when animals were exposed to DBP during the full treatment window (e13.5–e20.5) or during the MPW (e15.5–e18.5), but not after exposure during the late treatment window (e19.5–e20.5, after the MPW). Exposure to Dex modestly reduces Leydig cell steroidogenic function and AGD, but does not result in Leydig cell aggregates, demonstrating that such effects can occur in the absence of detectable dysgenesis. When large Leydig cell aggregates occur they are usually part of focal dysgenetic areas, which are abnormal in their cellular composition (eg presence of ectopic Sertoli cells) and are associated with reduced Leydig cell function (see below). Furthermore, it is within these focal dysgenetic areas, seen in fetal life, that malformed seminiferous cords with intratubular Leydig cells can develop after birth [8], [33], as confirmed presently. The latter persist for life as Sertoli-cell only tubules [8], [33], which are also found commonly in adult men with testicular germ cell cancer [27] as well as in some men with low sperm counts [28] and cryptorchidism [34].

Demonstration of the MPW, within which androgens must act to ensure correct later development of the male reproductive tract, led to the hypothesis that AGD measurements can be used as an indicator of fetal androgen production/exposure during this developmental time window and therefore as a ‘predictor’ of adult-onset male reproductive disorders [10], [14], [15], [30], [32]. Similar to rat studies, shorter AGD in human males is associated with occurrence of hypospadias, cryptorchidism and shorter penis length at birth [22], [23] and with low sperm counts and infertility in adulthood [24], [25]. The present studies confirm and extend previously published data [15], [31], by showing that, irrespective of when the *in utero* treatments are applied and when ITT is reduced, AGD only reflects effects within the MPW as only treatments including the MPW significantly reduced AGD in e21.5 fetuses. Therefore, AGD provides a robust ‘read-out’ of somatic (Leydig) cell function *specifically* in the MPW.

In this study we found a strong negative correlation between the degree of testicular dysgenesis and AGD at both e21.5 and postnatal day (pnd) 8. This relationship was independent of the timing or duration of DBP treatment, and suggests that dysgenesis and Leydig (somatic) cell function during the MPW are closely interlinked, consistent with a common cause/origin. Our results point to this relationship occurring because of a common cause rather than the induction of dysgenesis secondarily causing impaired Leydig cell function. First, the Leydig cell impairment is evident before dysgenesis is apparent. Second, impairment of Leydig cell function was induced by late window DBP treatment, which did not induce dysgenesis and, conversely, DBP treatment in the MPW caused dysgenesis and impaired Leydig cell function in the MPW (as indicated by AGD) whereas Leydig cell function had largely recovered by e21.5 following cessation of DBP treatment. This interpretation is strongly supported by the correlation analyses between ITT at e21.5 and either dysgenesis or AGD. Both showed that a strong correlation was only evident when data was included for animals in which DBP ± Dex exposure was maintained throughout (from e13.5–e20.5), and disappeared when animals were included that had been exposed only in early (MPW) or late time windows. This implies that when DBP exposure induces changes that lead to both dysgenesis and impaired Leydig cell function in the MPW, the latter but not the former is able to largely recover if treatment ceases at the end of the MPW. This also shows that measurement of testosterone levels at one stage in development is not necessarily reflective of levels at another time. Conversely, if DBP treatment continues after the MPW the impairment of Leydig cell function is maintained. Partial or complete recovery of Leydig cell function after cessation of DBP/phthalate treatment has been described previously by us [31] and others [35]. Aside from Pnd8, all of our correlation analyses were performed at the same age (e21.5), as this is the earliest age at which all relevant parameters could be measured simultaneously. Despite this, our findings imply that both AGD and dysgenesis provide read-outs of events several days earlier in gestation, namely during the MPW, and that both appear to mark a common event. Exactly what this is, is unknown, but it seems likely that a common, DBP-sensitive, mechanism that leads to impaired Leydig cell function (at any fetal age) also leads to dysgenesis, perhaps by affecting an aspect of cell lineage specification, consistent with the abnormal mixture of somatic (Sertoli, Leydig) cells found in some focal dysgenetic areas at e21.5 in DBP-exposed animals.

This study has implications for human male reproductive health. First, it provides strong support for the TDS hypothesis as the data suggest that early set-up problems (during the MPW) most likely result in effects (somatic cell dysfunction) that lead to TDS disorders in later life. Focal testicular dysgenesis is simply a reflection of poor set-up during this early programming period, even though it is not observed itself until some time later in development. Therefore, if the present experimental studies in rats can be extrapolated to humans, they imply that occurrence of areas of focal dysgenesis in humans, which can include at least some cases of Sertoli-cell-only (SCO) tubules [27], [28], may be a visible indicator of deficient androgen production/action in the MPW. If this is the case then reduced AGD in humans should correlate with the degree of testicular dysgenesis and this could be investigated in men with TGCC or who are being biopsied for low sperm counts. Two studies have already shown that shorter AGD is associated with low sperm counts and infertility in adulthood [24], [25], consistent with this thinking. Second, the current experimental studies suggest that there is an early, unknown, event, which can be disturbed by exposure to DBP, which fundamentally alters set-up of testis development and also impairs Leydig cell function. Our DBP studies in the rat can be used to identify this mechanism and investigate what factors, in addition to DBP, might impact this mechanism, as these would be likely to cause TDS disorders in human males.

This study and the human studies mentioned above clearly demonstrate the importance of early fetal life for later male reproductive health and function. We have demonstrated that exposing animals *in utero* to a treatment that disrupts normal testis development and causes focal testicular dysgenesis also impairs Leydig cell function during the MPW, which can lead to later TDS disorders but which is also captured by altered AGD. We believe that AGD in newborn boys therefore provides a reliable ‘read-out’ of androgen production/action during the MPW, and perhaps also of dysgenesis. We further hypothesize that AGD at birth can indicate whether a boy is susceptible to develop adult-onset TDS disorders, such as low sperm counts and TGCC.

## Materials and Methods

### Animals and treatments

Wistar rats were maintained according to UK Home Office guidelines (which also involves an ethical approval step) and were fed a soy-free breeding diet (RM3(E) soya free; SDS, Dundee, Scotland). Housing conditions were carefully controlled (lights on at 0700, off at 1900 h, temperature 19–21 C, GOLD shavings and LITASPEN standard bedding (SPPS, Argenteuil, France)). Time-mated female rats were subjected to the daily treatments described below, which were administered between 0900 and 1030 h. Three different treatment windows were used in this study for animals that were to be sampled on embryonic day (e) 21.5 (Fig. 1), namely “full treatment window” (e13.5–e20.5), “MPW treatment window” (e15.5–e18.5) and “late treatment window” (e19.5–e20.5). The doses of dibutyl phthalate (DBP) and dexamethasone (Dex) were based on previous studies [5], [30], [36], but in order to induce a higher level of testicular dysgenesis, 750 mg/kg of DBP was used in addition to the more common used dose of 500 mg/kg, which had previously been shown to induce focal dysgenetic areas in ∼60% of animals [5], [8]. The DBP was 99% pure according to the supplier. Rat treatment groups were as follows:

1. DBP (Sigma-Aldrich Co. Ltd., Dorset, UK) at a dose of either 500 or 750 mg/kg administered by oral gavage in 1 ml/kg corn oil.
2. Dex (Sigma-Aldrich) at a dose of 100 µg/kg by subcutaneous injection in 1 ml/kg saline.
3. A combination of DBP (500 mg/kg by oral gavage) plus Dex (100 µg/kg subcutaneously).
4. Control (1 ml/kg corn oil by gavage and 1 ml/kg saline by subcutaneous injection).

In a separate study, time-mated female rats were treated with Dex (100 µg/kg by subcutaneous injection), DBP (500 mg/kg by oral gavage) or a combination of DBP (500 mg/kg)+Dex (100 µg/kg) or vehicle from e13.5–e21.5 with termination at postnatal day (pnd) 8.

### Tissue recovery and processing

To acquire fetal samples, dams were killed by inhalation of CO_2_ followed by cervical dislocation at e21.5. Fetuses were removed, weighed, decapitated and placed in ice cold phosphate buffered saline (PBS; Sigma-Aldrich). Pnd8 pups were killed by inhalation of CO_2_ followed by cervical dislocation. AGD was measured before opening of the abdomen, using digital calipers (Faithfull Tools, Kent, UK). Testes were microdissected and weighed. One testis was fixed in Bouin's fixative for 1 hour at room temperature while the other testis was snap frozen on dry ice and stored at −70°C for determination of intratesticular testosterone (ITT) as described previously [5]. The limit of detection of the testosterone assay was 40 pg. Bouin's-fixed tissues were processed and embedded in paraffin wax, and 5-µm sections were used for subsequent experiments.

### Immunohistochemistry for 3β-hydroxysteroid dehydrogenase

In order to visualize Leydig cell aggregates in the collected testes, immunohistochemistry for 3β-hydroxysteroid dehydrogenase (3β-HSD) was performed on a Leica BOND-MAX automatic immunostaining machine using the BOND Polymer Refine Detection (Leica, UK). The 3β-HSD antibody (Santa Cruz Biotechnology, Inc., CA, USA) was diluted 1∶750.

### Double immunohistochemistry for 3β-HSD and Smooth Muscle Actin

In order to delineate the seminiferous cord compartment from the interstitial compartment and to visualize focal dysgenesis at postnatal day (pnd) 8, specific antibodies were used for the co-immunolocalization of α-smooth muscle actin (α-SMA; Sigma-Aldrich) and 3β-HSD (Santa Cruz Biotechnology) as described by Hutchison et al. [33].

### Double immunofluorescence for 3β-HSD and Sox-9

In order to visualize focal dysgenesis, specific antibodies were used for co-immunolocalization of 3β-HSD (Leydig cell marker; Santa Cruz Biotechnology) and Sox-9 (Sertoli cell marker; Chemicon International, UK). All washes between incubation steps were in TBS (3×5 min) and all incubations were carried out in a humidity box (Fisher Scientific, UK). Sections were dewaxed and rehydrated, followed by a peroxidase block in 3% (v/v) H_2_O_2_ in methanol for 30 min. Next, the sections were blocked in normal chicken serum (NCS; Biosera, Ringmer, UK) diluted 1∶5 in TBS containing 5% (w/v) BSA (NCS/TBS/BSA), followed by incubation with anti-Sox-9 antibody diluted 1∶5,000 in NCS/TBS/BSA overnight at 4°C. The next day, sections were incubated with peroxidase-conjugated chicken anti-rabbit secondary antibody (CARP; DAKO Corp., Cambridge, UK), diluted 1∶200 in NCS/TBS/BSA for 30 minutes at room temperature (RT), and followed by incubation with Tyr-Cy3 (Perkin Elmer-TSA-Plus Cyanine3 System; Perkin Elmer Life Sciences, Boston, MA, USA) according to the manufacturer's instructions. Sections were then subjected to antigen retrieval by boiling in a pressure cooker in 0.01 mol/l citrate buffer (pH 6.0) for 5 min and left to cool for 20 minutes, followed by another block in NCS/TBS/BSA and overnight incubation at 4°C with anti-3β-HSD antibody diluted 1∶6,000 in NCS/TBS/BSA. On the third day, slides were incubated with peroxidase-conjugated chicken anti-goat secondary antibody (Sigma-Aldrich) diluted 1∶200 in NCS/TBS/BSA for 30 minutes at RT, followed by incubation with Tyr-Cy5 (Perkin Elmer-TSA-Plus Cyanine5 System; Perkin Elmer Life Sciences) according to the manufacturer's instructions. Slides were counterstained for 10 minutes using 4′,6-diamidino-2-phenylindole (DAPI; Sigma-Aldrich) diluted 1∶1,000 in TBS. Finally, the slides were mounted with Permafluor (Thermo Scientific, UK) and fluorescent images were captured using a Zeiss LSM 710 Axio Observer Z1 confocal laser microscope (Carl Zeiss Ltd.).

### Measurement of Leydig cell aggregation

Measurement of Leydig cell aggregate size in the fetal testis after DBP ± Dex treatment was done as described previously [8]. Briefly, testes from the different treatment groups (n = 8–15 from 3–5 litters per treatment group) were serially sectioned and three representative sections from each testis then selected and immunostained for 3β-HSD. The three sections chosen were those corresponding to approximately 25, 50, and 75% intervals through the serially sectioned testis. Sections immunostained for 3β-HSD were not counterstained, so as to provide sufficient homogeneity, high contrast, and low background to allow computer-assisted thresholding and subsequent computer-assisted counting of Leydig cell (3β-HSD-immunopositive) aggregates and determination of Leydig cell aggregation area. This was done using a Zeiss Axio-Imager microscope (Carl Zeiss Ltd., Welwyn Garden City, UK) fitted with a Hitachi HV-C20 camera (Hitachi Denshi Europe, Leeds, UK) and Image-Pro 6.2 software (MagWorldwide, Wokingham, UK). The software was used to trace around each section, creating an area of interest, allowing the area of each section to be calculated. Computer-assisted thresholding was then used to identify and analyze aggregates or clusters of 3β-HSD-immunopositive cells, generating data on aggregate area and the proportion of each section occupied by Leydig cell aggregates. Leydig cell aggregates were then assigned arbitrarily to one of three groups: small aggregates, accounting for ≤5% of the total Leydig cell aggregate area per testis, and large aggregates, which individually accounted for ≥5.1% of total Leydig cell aggregate area per testis.

For quantification of Leydig cell aggregates at pnd8, two sections per animal (n = 8–17 from 3–6 litters per treatment group) were stained with 3β-HSD as described above. Because of the size of the testes and the infrequency of fetal Leydig cells at this age, the same analysis as done for the fetal samples was not practical. Therefore, the three largest Leydig cell aggregates from each section were selected visually and then measured as described above. The mean of the measured Leydig cell aggregates per testis per animal was then calculated and used for analysis.

### Statistical analysis

Values are expressed as mean ± SEM. Comparison of treatment effects used one-way ANOVA followed by the Bonferroni post test, whereas linear regression analysis was used to determine the relationship between AGD and Leydig cell aggregation or ITT. Data for ITT was log transformed prior to analysis to normalize distribution and variance. These analyses used GraphPad Prism (version 5; GraphPad Software Inc., San Diego, CA). The presented data used each animal as the unit, rather than the litter, because the basis of the present studies was to evaluate the inter-relationships between treatment effects on AGD, ITT and dysgenesis at the individual level.

## Supporting Information

Figure S1 **Contribution of small and large Leydig cell aggregates to the total Leydig cell aggregate area per testis in e21.5 rat testes after** ***in utero*** **exposure to vehicle (control) or dibutyl phthalate (DBP-500 or 750 mg/kg), dexamethasone (Dex 100 µg/kg) or DBP-500+Dex from e13.5–e20.5 (full treatment window), e15.5–e18.5 (MPW window) or e19.5–e20.5 (late window) analyzed as litter means.** Values are Means ± SEM for 3–5 litters per treatment group. ***p<0.001, in comparison with controls; ^a^p<0.001 in comparison with Dex group (except p<0.05 when Dex is compared with DBP-500 late window treatment); ^b^p<0.05 in comparison with DBP-500 late window group; ^c^p<0.001 in comparison with DBP-750 late window group; ^d^p<0.05 in comparison with DBP-750 full treatment window group.
(TIF)

Figure S2 **Litter means of anogenital distance (AGD) and intratesticular testosterone (ITT) in rats at e21.5 after** ***in utero*** **exposure to vehicle (control), dibutyl phthalate (DBP-500 or 750 mg/kg), dexamethasone (Dex 100 µg/kg) or DBP-500+Dex from e13.5–e20.5 (full treatment window), e15.5–e18.5 (MPW window) or e19.5–e20.5 (late window).** Values are Means ± SEM for 3–7 litters per group. *p<0.05, **p<0.01, ***p<0.001, in comparison with controls; ^a^p<0.001 in comparison with Dex group; ^b^p<0.001 in comparison with DBP-500 late window group; ^c^p<0.001 in comparison with DBP-750 late window group.
(TIF)

Figure S3 **Relationship between Leydig cell (LC) aggregation ( = focal dysgenesis) and anogenital distance (AGD) (A, B) or intratesticular testosterone (ITT) at e21.5 (C, D) or between AGD and ITT at e21.5 (E, F) in animals exposed** ***in utero*** **to vehicle (control), dibutyl phthalate (DBP-500 or 750 mg/kg), dexamethasone (Dex 100 µg/kg) or DBP-500+Dex during all treatment windows (A, C, E), or during the full treatment window (e13.5–e20.5) only (B, D, F), analyzing the data as litter means.**
(TIF)
