# Supplementary figures and images for: Inter-Relationship between Testicular Dysgenesis and Leydig Cell Function in the Masculinization Programming Window in the Rat
Source: PLoS One. 2012 Jan 11;7(1):e30111. doi: 10.1371/journal.pone.0030111 (PMC3256232; doi:10.1371/journal.pone.0030111)

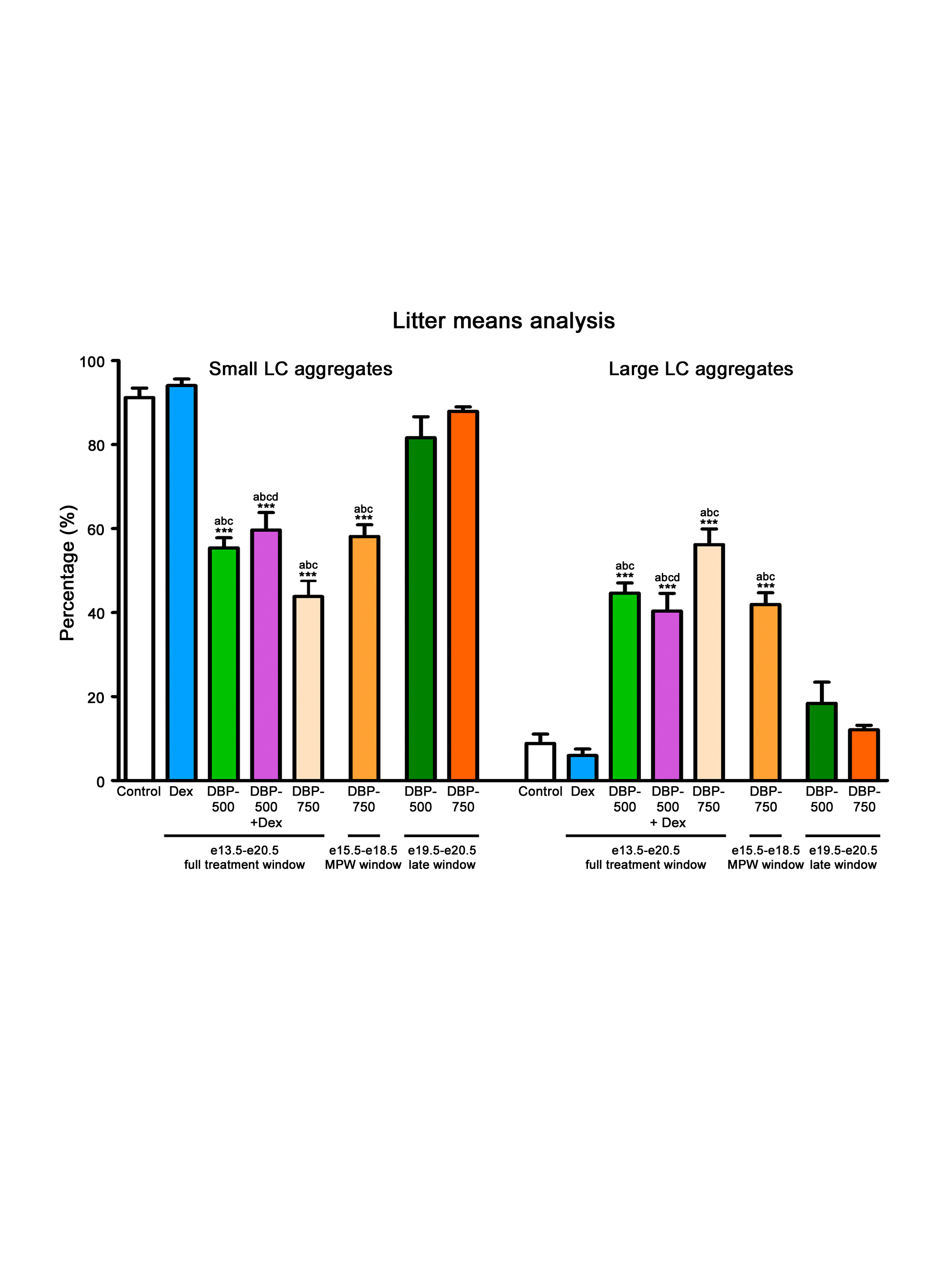

Supplement: Figure S1 — Contribution of small and large Leydig cell aggregates to the total Leydig cell aggregate area per testis in e21.5 rat testes after in utero exposure to vehicle (control) or dibutyl phthalate (DBP-500 or 750 mg/kg), dexamethasone (Dex 100 µg/kg) or DBP-500+Dex from e13.5–e20.5 (full treatment window), e15.5–e18.5 (MPW window) or e19.5–e20.5 (late window) analyzed as litter means. Values are Means ± SEM for 3–5 litters per treatment group. ***p<0.001, in comparison with controls; ap<0.001 in comparison with Dex group (except p<0.05 when Dex is compared with DBP-500 late window treatment); bp<0.05 in comparison with DBP-500 late window group; cp<0.001 in comparison with DBP-750 late window group; dp<0.05 in comparison with DBP-750 full treatment window group. (TIF) [file pone.0030111.s001.tif]

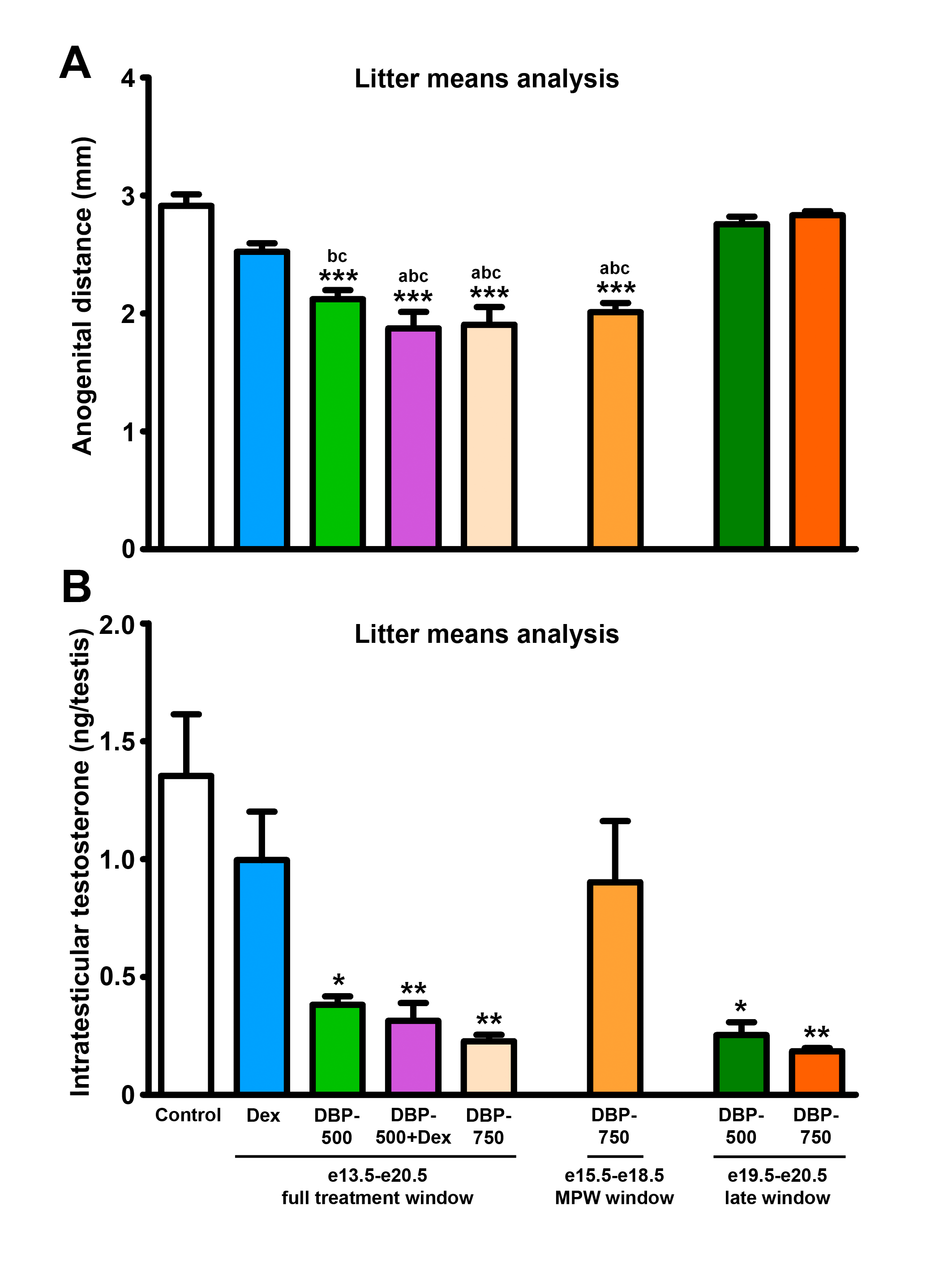

Supplement: Figure S2 — Litter means of anogenital distance (AGD) and intratesticular testosterone (ITT) in rats at e21.5 after in utero exposure to vehicle (control), dibutyl phthalate (DBP-500 or 750 mg/kg), dexamethasone (Dex 100 µg/kg) or DBP-500+Dex from e13.5–e20.5 (full treatment window), e15.5–e18.5 (MPW window) or e19.5–e20.5 (late window). Values are Means ± SEM for 3–7 litters per group. *p<0.05, **p<0.01, ***p<0.001, in comparison with controls; ap<0.001 in comparison with Dex group; bp<0.001 in comparison with DBP-500 late window group; cp<0.001 in comparison with DBP-750 late window group. (TIF) [file pone.0030111.s002.tif]

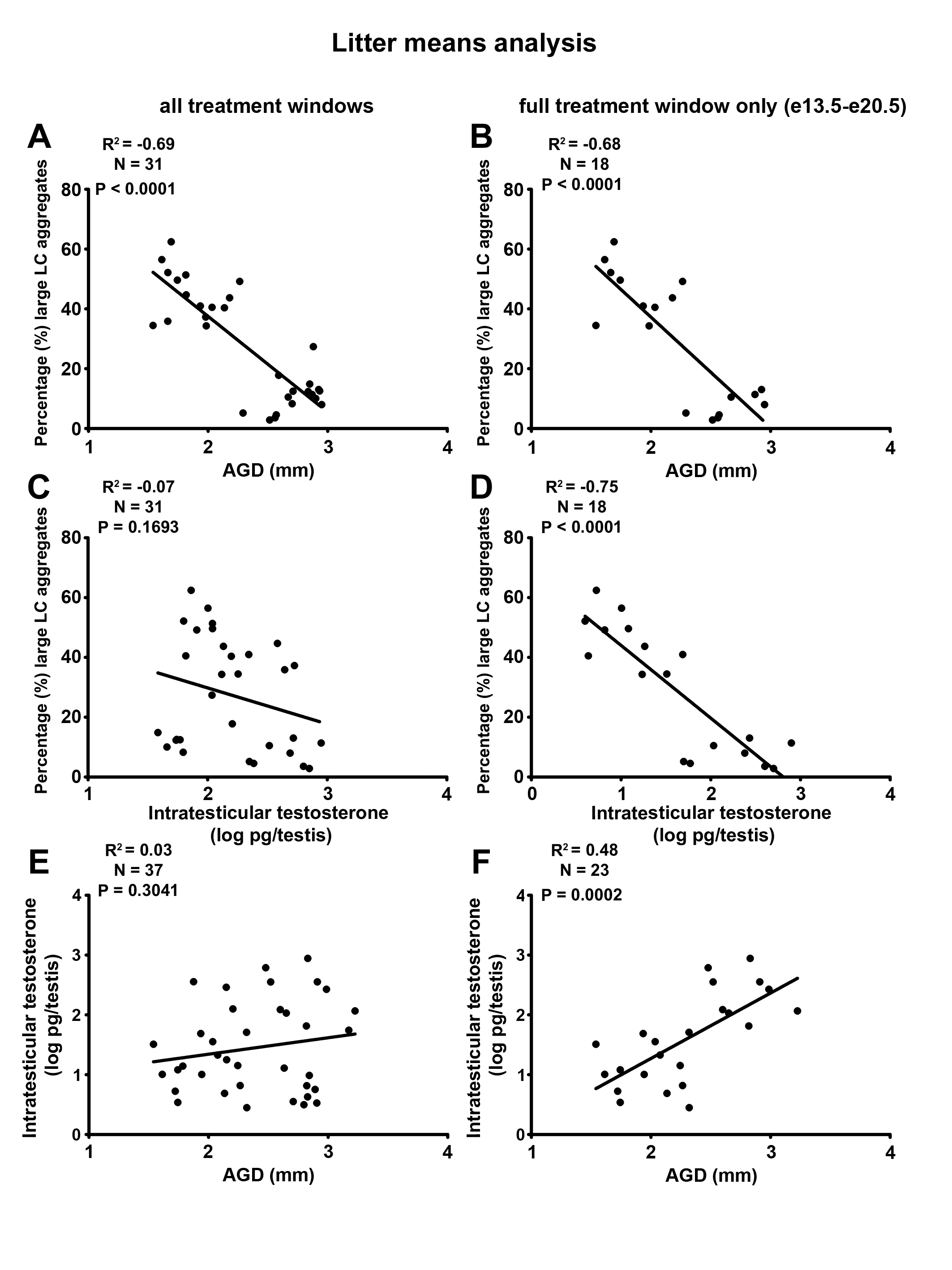

Supplement: Figure S3 — Relationship between Leydig cell (LC) aggregation ( = focal dysgenesis) and anogenital distance (AGD) (A, B) or intratesticular testosterone (ITT) at e21.5 (C, D) or between AGD and ITT at e21.5 (E, F) in animals exposed in utero to vehicle (control), dibutyl phthalate (DBP-500 or 750 mg/kg), dexamethasone (Dex 100 µg/kg) or DBP-500+Dex during all treatment windows (A, C, E), or during the full treatment window (e13.5–e20.5) only (B, D, F), analyzing the data as litter means. (TIF) [file pone.0030111.s003.tif]
